# Verbal adynamia in parkinsonian syndromes: behavioral correlates and neuroanatomical substrate

**DOI:** 10.1080/13554794.2018.1527368

**Published:** 2018-10-06

**Authors:** Nadia K Magdalinou, Hannah L Golden, Jennifer M Nicholas, Pirada Witoonpanich, Catherine J Mummery, Huw R Morris, Atbin Djamshidian, Tom T Warner, Elizabeth K Warrington, Andrew J Lees, Jason D Warren

**Affiliations:** a Reta Lila Weston Institute of Neurological Studies, UCL Institute of Neurology, London, UK; b Dementia Research Centre, UCL Institute of Neurology, London, UK; c Department of Medical Statistics, London School of Hygiene and Tropical Medicine, London, UK; d Division of Neurology, Department of Medicine, Faculty of Medicine, Ramathibodi Hospital, Mahidol University, Bangkok, Thailand; e Department of Clinical Neuroscience, UCL Institute of Neurology, London, UK

**Keywords:** Parkinson’s disease, Lewy body disease, progressive supranuclear palsy, corticobasal degeneration, dynamic aphasia

## Abstract

Verbal adynamia (impaired language generation, as during conversation) has not been assessed systematically in parkinsonian disorders. We addressed this in patients with Parkinson’s dementia, progressive supranuclear palsy and corticobasal degeneration. All disease groups showed impaired verbal fluency and sentence generation versus healthy age-matched controls, after adjusting for general linguistic and executive factors. Dopaminergic stimulation in the Parkinson’s group selectively improved verbal generation versus other cognitive functions. Voxel-based morphometry identified left inferior frontal and posterior superior temporal cortical correlates of verbal generation performance. Verbal adynamia warrants further evaluation as an index of language network dysfunction and dopaminergic state in parkinsonian disorders.

## 1 Introduction

Neurodegenerative parkinsonian syndromes with cognitive impairment are heterogeneous, encompassing Parkinson’s disease dementia (PDD), dementia with Lewy bodies (DLB), progressive supranuclear palsy (PSP), and corticobasal syndrome (CBS). Although cognitive decline in these disorders is often clinically significant, their neuropsychological phenotypes remain incompletely defined. Linguistic deficits occur in PDD and DLB but have often been attributed to secondary effects of primary executive or working memory impairment (Kertesz, Martinez-Lage, Davidson, & Munoz, 2000). In contrast, nonfluent aphasia and speech apraxia are well-recognized presentations of CBS and PSP (Kertesz et al., 2000; Rohrer et al., 2010) and may signify a broader spectrum of linguistic dysfunction in this disease group. Whereas CBS is commonly associated with early speech production deficits overlapping with progressive nonfluent aphasia (Kertesz et al., 2000), Parkinson’s disease and DLB may be associated with deficits of sentence processing and verbal working memory (Gross et al., 2012). In principle, linguistic profiles might distinguish diseases with abnormal deposition of synuclein (synucleinopathies: PDD, DLB) (Spillantini et al., 1997) from those with abnormal deposition of tau (tauopathies: CBS, PSP) (Arai et al., 2001) and the heterogeneous other pathologies underpinning CBS and progressive nonfluent aphasia (Kertesz & McMonagle, 2010). However, the potential of neuropsychological and, in particular, linguistic profiles to differentiate parkinsonian syndromes remains largely unexplored.

A prominent feature of parkinsonian disorders is reduced self-generated initiation of motor actions, manifesting most clearly in motor akinesia (Bhatia & Marsden, 1994). This might plausibly have a linguistic analogue in deficits of verbal processes required to generate propositional speech. Here, we refer to speech as exemplified by everyday conversation, in which we are generally required to create a verbal message, however banal. Deficits of propositional speech output we subsume under the general term “verbal adynamia;” in its most well-defined and selective form, impaired generation of novel verbal messages leads to a characteristic, specific language disorder, “dynamic aphasia.” Dynamic aphasia is characterized by disproportionate impoverishment of propositional speech despite relatively preserved ability to produce speech in specific contexts such as naming, repetition, or reading; the core cognitive deficit has been variously held to reflect impaired construction of a sentence scheme, generation of novel verbal ideas, fluent sequencing of verbal thought, or selection among prepotent verbal alternatives (Costello & Warrington, 1989; Blank, Scott, Murphy, Warburton, & Wise, 2002; Esmonde, Giles, Xuereb, & Hodges, 1996; Robinson, Shallice, & Cipolotti, 2006; Wagner, Pare-Blagoev, Clark, & Poldrack, 2001; Warren, Warren, Fox, & Warrington, 2003). These formulations are not mutually incompatible and more than one cognitive mechanism may operate, particularly in the setting of strategic or diffuse brain damage (Esmonde et al., 1996; Robinson et al., 2006; Robinson, Spooner, & Harrison, 2015). Moreover, verbal adynamia may occur in milder form or accompanying other linguistic or executive deficits, as indexed by verbal fluency tasks. Verbal fluency deficits are well described in PD and are likely in part to reflect motor slowing together with executive compromise. Such deficits may also signify the involvement of language generation mechanisms and may help differentiate parkinsonian disorders, potentially while clinical disease is still mild (Petrova et al., 2016; Rittman et al., 2013).

Dynamic aphasia has been documented in association with various focal brain lesions involving the dominant frontal lobe and its connections (Blank et al., 2002; Braun, Guillemin, Hosey, & Varga, 2001; Robinson, 2013; Wagner et al., 2001) as well as neurodegenerative syndromes implicating this circuitry, notably PSP (Bak, Hodges, & Thomas, 2008; ; Esmonde et al., 1996; Litvan et al., 1996; Robinson, 2013; Robinson et al., 2015; Warren et al., 2003). Propositional speech production is mediated by a distributed neural network including left superior and inferior frontal gyri, frontal operculum, and posterior superior temporal cortex (Blank et al., 2002; Braun et al., 2001; Wagner et al., 2001). Disruption of these fronto-subcortical networks and, more particularly, dopaminergic projection pathways is clearly a candidate substrate for development of verbal adynamia across parkinsonian disorders (Robinson, 2013). However, disentangling the role of motor, executive, and primary linguistic impairments in the context of parkinsonism is challenging. Moreover, the neuroanatomical basis of neurolinguistic and related cognitive impairments in the parkinsonian spectrum and the potentially modulatory effects of dopaminergic therapy remain incompletely defined.

In this study, we set out to assess and compare verbal adynamia (as a critical component of natural communication) in major parkinsonian syndromes. Our main objectives were, first, to provide evidence that this important function can be isolated as a locus of impairment in these syndromes once other potentially relevant (especially, executive) processes are taken into account; second, to assess the modulatory effect of dopaminergic activity on verbal adynamia, given the central pathophysiological role of dopaminergic deficiency in parkinsonism; and third, to link verbal aydnamia to an underlying neuroanatomical substrate in these disorders. We assessed generation of novel verbal output in relation to other linguistic, executive, and general cognitive functions, in cohorts of patients with PDD, PSP, and CBS. We compiled a neuropsychological battery to assess verbal adynamia in each of these syndromes, by probing both general verbal fluency and generation of more extended verbal output. In addition, we used this battery to assess the effects of dopaminergic modulation on generation of verbal output in PDD. As part of the neuropsychological assessment, we adjusted for the effects of extra-linguistic executive and associated linguistic deficits, in order to disambiguate verbal adynamia from potentially related or confounding deficits. We assessed neuroanatomical associations of verbal generation using voxel-based morphometry (VBM) of patients’ brain MR images. We hypothesized firstly that verbal adynamia (defined as a specific linguistic disorder affecting the generation of novel verbal material, disproportionately or independently of other cognitive deficits) would be a feature of each of these parkinsonian syndromes. More specifically, we hypothesized that patients would have greater difficulty on tasks requiring generation of verbal material *de novo* (such as producing a word list or completing open-ended sentences) than tasks in which the verbal program was constrained by context (such as completing sentences with a conclusion strongly implied by context); and greater difficulty generating more extended verbal messages (phrases and sentences) *de novo* than completing sentences with single words. Second, we hypothesized on clinical and neuroanatomical grounds that verbal adynamia (as an index of reduced processing flexibility in fronto-subcortical brain networks) would differentiate parkinsonian syndromes: we predicted that verbal generation deficits would be more severe in PSP and CBS than in PDD, but would also show greater sensitivity to dopaminergic modulation than other cognitive deficits in PDD. Finally, we hypothesized a neuroanatomical correlate of verbal adynamia in dominant hemisphere circuits previously implicated in the generation of propositional speech (Blank et al., 2002; Braun et al., 2001).

## 2 Methods

### 2.1 Participants

Eighteen consecutive patients with PDD, 7 patients with PSP, and 4 patients with CBS were identified via the National Hospital for Neurology and Neurosurgery specialist clinics over a 12-month period. All patients fulfilled current consensus criteria for the relevant diagnosis (Armstrong et al., 2013; Emre et al., 2007; Hoglinger et al., 2017); none presented with speech or language disturbance as the leading clinical feature. In addition, all had a history of cognitive symptoms significant enough to interfere with everyday functioning with previous documentation of objective cognitive impairment on clinical neuropsychometry. Severity of cognitive and motor impairment ranged from mild to moderate over the cohort (see Table 1). Patients are hereafter grouped as PDD and PSP/CBS, respectively, to reflect the pathological separation between presumptive primary synucleinopathies and other proteinopathies (particularly tauopathies). Nineteen healthy age-matched individuals with no history of psychiatric or neurological disease were also included. All participants were native English speakers.

**Table 1.** Summary of group demographic, clinical, and background neuropsychological data.

Characteristic	Controls	PDD	PSP/CBS
***General***			
Number (female:male)	19 (13:6)	18 (4:14)**	11 (7:4)*
Age (years)	70 (6.2)	72 (6.4)	69 (7.3)
Handedness (right:left)	18:1	17:1	11:0
Education (years)	15 (3.6)	13 (2.8)	15 (3.2)
***Clinical***			
Symptom duration (years) (median (IQR))	NA	10 (7, 14)	3 (2, 5)**
MMSE/30 mean (range)	NA	25 (24–26)**	27 (24–28)
HY (mean (*SD*))	NA	3 (0.9)	3 (1.1)
UPDRS (mean (*SD*))	NA	37 (10)	30 (9.2)
***General cognitive level***			
Verbal IQ raw score (percentile rank)	120 (91)	106 (63) *	88 (20) **
Performance IQ raw score (percentile rank)	120 (91)	85 (16) *	85 (16) *
**VERBAL skills**			
**Word knowledge**			
Concrete synonyms (Warrington & Orpwood, L., 1998)	>75	<50*	<50*
Abstract synonyms (Warrington & Orpwood, L., 1998)	>75	<50*	<50*
**Reading, writing, and spelling**			
NART (Nelson, 1982) predicted verbal IQ	117	113	109
Baxter spelling test (Baxter & Warrington, 1994)	>75	>50*	>50*
**EXECUTIVE skills**			
WASI matrices(Lange, Chelune, & Tulsky, 2006)	>75	<25*	<25*
WASI block design(Lange et al., 2006)	>75	<25*	<50*
WASI similarities(Lange et al., 2006)	>75	<50*	<50*
**EPISODIC memory**			
RMT-Faces (Warrington, 1984)	>50	<25	<10
RMT-words (Warrington, 1984)	>75	<10*	<25*
**OTHER skills**			
Graded difficulty arithmetic (Jackson & Warrington, 1986)	>50	<10*	<10*
VOSP Object Decision (Warrington & James, 1991)	>50	<5*	<5*

Percentile rank values are shown unless otherwise indicated. *significantly lower than healthy control group; **significantly lower than other disease group; HY, Hoehn and Yahr score; IQR, inter-quartile range; IQ, intelligence quotient; MMSE, Mini-Mental State Examination score; NA, not applicable; NART, National Adult Reading Test; PDD, patient group with Parkinson’s disease dementia; PSP/CBS, patient group with progressive supranuclear palsy/corticobasal syndrome; RMT, Recognition Memory Test; SD, standard deviation; UPDRS, Unified Parkinson’s Disease Rating Scale; VOSP, Visual Object and Spatial Perception battery; WASI, Wechsler Abbreviated Scale of Intelligence.

Ethical approval for the study was obtained from the local institutional ethics committee and all participants gave written informed consent in accordance with the Declaration of Helsinki.

### 2.2 General disease assessments

Patients had a comprehensive clinical assessment including Hoehn and Yahr (Hoehn & Yahr, 1967) staging and Unified Parkinson’s Disease Rating Scale (UPDRS) (Movement Disorder Society Task Force on Rating Scales for Parkinson’s Disease, 2003). Median symptom duration was 10 years in the PDD group and three years in the PSP/CBS group. All PDD patients were receiving dopaminergic therapy at the time of assessment (further details in Table S1 in Supplementary Material online) and were experiencing moderate but definite motor fluctuations with clear ON and OFF states (see Table S2 in Supplementary Material online); most (12/18) patients with PDD were also taking an acetylcholinesterase inhibitor for management of cognitive symptoms. No patients in the PSP/CBS group were taking dopaminergic or acetylcholinesterase inhibitor medications.

All participants had a comprehensive general neuropsychological assessment including executive, language, episodic memory, and posterior cortical functions (summarized in Table 1; tests described in Supplementary Material online). Three patients with CBS underwent lumbar puncture as part of their clinical work-up; none of these had a CSF profile suggesting underlying Alzheimer pathology, as evidenced by a total tau: beta-amyloid_1-42_ ratio <0.5 in each case.

### 2.3 Assessment of verbal adynamia

Verbal dynamic function was investigated using a series of tasks designed to sample verbal generation in various contexts, on scales ranging from single words to complete sentences. In order to provide overall measures of verbal fluency, we administered a letter fluency task requiring generation of words from a nominated initial letter and a category fluency task requiring generation of words according to a specific semantic criterion (kinds of animals). The total number of words produced in 60 s was scored in each case. In addition, tests were administered requiring participants either to complete or continue a linguistic stimulus in order to create a verbal message (tasks adapted from previous work (Costello & Warrington., 1989), (Bloom & Fischler, 1980), (Wechsler, 1991), listed in Table 2; stimuli in Table S3 in Supplementary Material online). In the first of these tasks, short written sentences were presented requiring completion with a single word that was either strongly determined by context (highly predictable or “constrained” completions, of the form: “He loosened the tie around his…”) or not strongly determined by context (less predictable or “unconstrained” completions, of the form: “Jack went to the shop to buy a…”); 10 constrained and 10 unconstrained trials were presented consecutively, as separate blocks. In order to assess generation of more extended verbal output, we used tasks requiring completion of a sentence stem with a phrase (of the form, “The children were…”; eight trials) or generation of a sentence from a sentence context (of the form, “Joe had fallen and twisted his ankle.”; eight trials). Finally, we designed a test to assess generation of a verbal message *de novo*: participants were presented with a sequence of three line drawings and required to generate a sentence describing what might happen next (see examples in Supplementary Figure S1 online; six trials). Participants responded verbally and responses were recorded for offline analysis. Correct responses were semantically congruent and grammatically correct (in the case of sentences, these had to contain a subject and predicate with a verb). Examples of grammatical and semantic errors are provided with Figure S1; no response by 20 s was also recorded as an error for that trial.

**Table 2.** Summary of participant group performance on verbal adynamia battery.

Cognitive function	Controls	PDD	PSP/CBS
**VERBAL generation functions**			
Verbal fluency: Initial letter (in 60 secs)	17 (12–26)	9 (3–17)*	7 (1–13)**
Verbal fluency: category (in 60 secs)	21 (18–24)	11 (7–13)*	9 (7–12)*
Sentence completion: constrained word (/10)	10	10 (8–10)	10 (8–10)
Sentence completion: unconstrained word (/10)	10 (8–10)	9 (8–10)	9 (6–10)
Sentence completion: phrase (/8)	8	7 (6–8)	8 (3–8)
Sentence generation: from sentence (/8)	8 (5–8)	5 (3–7)*	4 (2–8)*
Sentence generation: from picture sequence (/6)	6 (5–6)	3 (2–6)*	5 (1–6)*
**CONTROL FUNCTIONS**			
Nonverbal fluency: design (Kaplan, 2001) (in 60 secs)	10 (4–17)	6 (2–10)*	4.5 (3–7)*
Naming: GNT (McKenna & Warrington, 1983) (/30) (mean, *SD*)	24 (4.1)	21 (4.6)*	17 (8.1)*
Naming: BNT (Kaplan & Weinthaubs S., 2001) (/30) (mean, *SD*)	28 (2.5)	26 (3.2)	23 (6.0)*
Naming: Verbs (Rohrer et al., 2010) (/20)	20 (20–20)	20 (17–20)*	19 (14–20)*
Expressive grammar (Helm-Estabrooks et al., 2000) (/12)	12 (11–12)	11 (9–12)*	11 (11–12)*
Receptive grammar-PALPA55 (Kay et al., 1992) (/24)	24 (21–24)	22 (15–24)*	22 (15–23)*

Maximum subtest scores are indicated where appropriate (in parentheses); median (range) data values are shown unless otherwise indicated. *significantly lower than healthy control group. **significantly lower than the PDD group. BNT, Boston Naming Test; GNT, Graded Naming Test; PALPA, Psycholinguistic Assessment of Language Processing in Aphasia; PDD, patient group with Parkinson’s disease dementia (here data for OFF state are presented); PSP/CBS, patient group with progressive supranuclear palsy/corticobasal syndrome; SD, standard deviation.

These verbal generation tasks were assessed together with measures of design fluency, noun and verb naming, and grammar processing (see Table 2), in order to determine the extent to which any impairment of verbal message generation was separable from other deficits of nonverbal generation, word retrieval, and sentence processing. The Delis-Kaplan Executive Function System design fluency test (Kaplan, 2001) was used to measure the participant’s ability to draw as many different designs as possible in 60 s; rows of boxes (each containing the same array of dots) were presented and the participant was asked to draw a different design in each box using only four straight lines to connect the dots. Noun retrieval was assessed using standard tests of picture naming (Graded Naming Test: (McKenna & Warrington, 1983), Boston Naming Test (Kaplan & Weinthaubs S., 2001)). Verb retrieval was assessed using a verb naming task adapted from Rohrer et al, 2010 (Rohrer, Crutch, Warrington, & Warren, 2010); participants were given 20 action pictures and were asked to name the relevant verbs. Grammar processing was assessed using a standard test of sentence comprehension derived from subtest 55 of the Psycholinguistic Assessment of Language Processing in Aphasia (Kay, Lesser, & Coltheart, 1992) (24 trials); and a test of expressive grammar derived from the Sentence Production Program for Aphasia (Helm-Estabrooks, Nicholas, & Helm, 2000), in which sentences embodying different grammatical constructions were elicited based on a picture accompanied by a brief story (12 trials).

The examiner ensured that participant understood each task using two initial practice trials that were not included in the analysis. No feedback about performance was given during the test.

### 2.4 Effect of dopaminergic medication

In order to assess the impact of dopaminergic modulation of verbal dynamic functions, a subset of 16 patients with PDD was assessed in both ON and OFF states on 2 consecutive days, using parallel versions of the relevant tests (stimulus sets A and B; see Table S3) to reduce practice effects. Linguistic functions assessed under dopaminergic modulation comprised letter fluency (“P” for stimulus set A and “S” for stimulus set B), sentence completion, phrase and sentence generation, design fluency, and expressive grammar. Motor features defining the shift between ON and OFF states were documented using the UPDRS part III (Disease, 2003). During the ON state (Day 1), patients were assessed 60 min after taking their usual anti-parkinsonian medications using stimulus set B; during the OFF state (Day 2), patients were assessed before taking their morning anti-parkinsonian medications, at least 12 h after the last medication dose, using stimulus set A. A subset of healthy control participants was also assessed on both stimulus sets A and B (administered several hours apart) in order to provide a reference to assess test—retest reproducibility (summarized in Table S4 in Supplementary Material online). For purposes of group comparisons, performance data of PDD patients in the OFF state were used: this is the endogenous, unmedicated disease state and therefore more suitable for comparing the PDD and PSP/CBS groups.

### 2.5 Analysis of behavioral data

Statistical analyses were implemented using STATA® (version 12.1, StataCorp LLC, College Station, Texas, USA). To compare demographic and neuropsychological data between groups, unpaired *t*-tests were used for normally distributed data, and Mann-Whitney U/Wilcoxon rank-sum tests were used if normality assumptions were materially violated. Fisher’s exact tests were used to compare the distribution of categorical variables across groups.

Performance on verbal generation tasks was compared between participant groups using a linear regression model, covarying for potentially confounding (nuisance) factors of gender and linguistic control task performance (lexical retrieval, Graded Naming Test score; sentence assembly, expressive grammar score). Verbal generation is likely a priori to be closely aligned to other cognitive control and generative processes: for the purposes of the present analysis, we incorporated WASI Matrices score (a standard measure of general nonverbal cognitive flexibility) as an additional nuisance covariate in the regression model. We did not attempt to adjust for design fluency (a measure of nonverbal pattern generation) in the same regression model but rather assessed correlation between verbal and nonverbal generation measures separately, as these measures might a priori be more closely and specifically associated (so covariation might therefore parcel out the effect of interest). All verbal adynamia battery data were non-normally distributed; accordingly, nonparametric, bias-corrected, and accelerated, bootstrap confidence intervals were calculated from 2,000 replications. In order to assess performance on the fluency tasks, scores were first converted into standardized z-scores based on mean (standard deviation) data from the healthy control group as a reference; paired *t*-tests were used to compare performance on different fluency tests in each participant group and two sample *t*-tests were used for comparison between groups. In order to assess the effect of dopaminergic medication on verbal adynamia battery performance in the PDD group, ON and OFF state data (for stimulus sets A and B) were compared using Wilcoxon signed-rank tests.

Correlations between MMSE score and performance on verbal adynamia and control tasks were assessed using Spearman’s rank correlation coefficient.

A statistical significance threshold *p* < 0.05 was accepted for all comparisons.

### 2.6 Brain image acquisition and analysis

At the time of behavioral assessment, 28 patients (18 PDD, 10 PSP/CBS) underwent volumetric T1-weighted brain MRI at 3 Tesla. After pre-processing of these MR images using New Segment (Weiskopf et al., 2011) and DARTEL (Ashburner, 2007) toolboxes of SPM8 (www.fil.ion.ucl.ac. uk/spm), regional gray matter correlates of verbal generation task performance were assessed across the combined patient cohort using generalized linear models implemented in a standard VBM protocol. Voxel intensity (gray matter volume) was modeled across the entire patient cohort as a function of letter fluency, sentence completion, and sentence generation test scores separately, incorporating total intracranial volume, gender, Graded Naming Test, and WASI matrices scores as covariates of no interest in each model. Gray matter associations of verbal adynamia control (naming, grammaticality) test performance were also assessed. To help protect against voxel drop out due to local regional atrophy, we applied a customized explicit brain mask based on a “consensus” voxel threshold intensity criterion (Ridgway et al., 2009).

Statistical parametric maps of regional gray matter volume associated with neurolinguistic scores were examined at local maximum threshold *p* < 0.05 after family-wise error (FWE) correction for multiple voxel-wise comparisons over the whole brain and after small volume correction within defined anatomical regions based on our prior anatomical hypotheses. Anatomical small volumes were derived from the Oxford–Harvard brain maps (Desikan et al., 2006) in FSL view (Jenkinson, Beckmann, Behrens, Woolrich, & Smith, 2012), comprising key brain regions previously implicated in generation of propositional language output (Blank et al., 2002; Braun et al., 2001; Wagner et al., 2001) (left inferior and superior frontal, left posterior superior temporal gyri).

## 3 Results

### 3.1 General participant group characteristics

The PDD and PSP/CBS groups were comparable in age, overall disability (Hoehn and Yahr), and motor impairment (UPDRS) (see Table 1). There was a gender imbalance between the groups (a higher proportion of males in the PDD group versus the PSP/CBS group) and gender was incorporated as a nuisance covariate in all analyses. The PDD group had significantly longer median symptom duration than the PSP/CBS group. Relative to the healthy control group, both disease groups showed significant deficits in a number of general cognitive domains including executive and working memory functions, verbal episodic memory, word knowledge, spelling, and posterior cortical functions. The PDD group had significantly lower mean Mini-Mental State Examination (MMSE) score than the PSP/CBS group; however, the absolute mean discrepancy between the patient groups was small (two MMSE points), with substantial overlap of scores between groups. There was no significant correlation between MMSE score and performance on any verbal adynamia or control task across the patient cohort.

### 3.2 Performance on verbal adynamia battery

**Figure 1.**
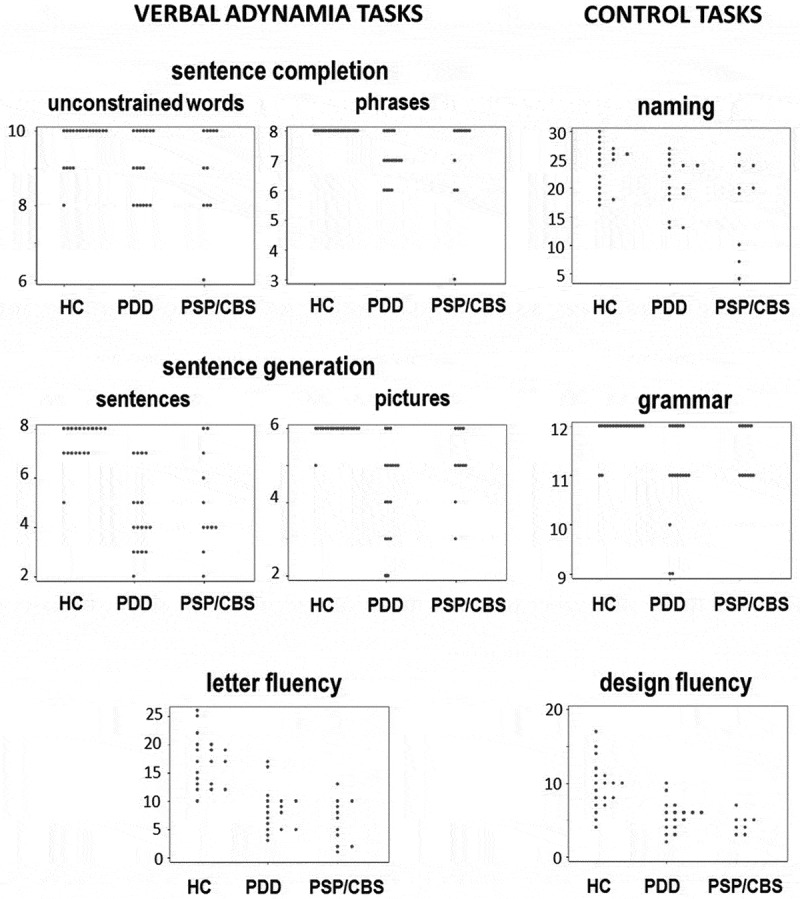
Scatter plots showing individual raw scores for all participant groups on tasks assessing verbal adynamia (sentence completion, sentence generation, letter fluency; see text for details) and on control tasks (Delis-Kaplan executive function system design fluency, Graded naming test, Sentence Production Program for Aphasia expressive grammar subtest). Note changes of vertical axis between tests. HC, healthy controls; PDD, patients with Parkinson’s disease dementia; PSP/CBS, patients with progressive supranuclear palsy/corticobasal syndrome.

Performance profiles of all participant groups on the verbal adynamia and control tasks are summarized in Table 2 and individual data are plotted in Figure 1; further details are in Tables S5, S6, and S7 in Supplementary Material online.

On the verbal adynamia control tasks, relative to the healthy control group both the PDD and PSP/CBS groups showed significant deficits of design fluency (*p* < 0.001) and in addition, significant deficits of naming and grammar processing (*p* < 0.05). There were no significant performance differences between patient groups on these control tasks. After taking performance on the verbal adynamia control tasks into account (by covarying for these in the regression model), relative to the healthy control group both the PDD and PSP/CBS groups had significantly reduced verbal (initial letter and category) fluency (*p* < 0.001) and performed significantly worse on sentence generation from a sentence context or cued by a picture sequence (*p* < 0.05). The patient groups did not show significant deficits of sentence completion relative to the healthy control group, though it is noteworthy that both patient groups showed a higher error rate and wider variation of performance on the unconstrained completions (Figure 1 and Table S5 online). There were no significant performance differences between patient groups on the verbal adynamia tasks (Table S6 online). There was no correlation between design fluency and verbal generation performance across the patient cohort (*Rs* 0.2–0.3).

Within each patient group, individuals varied widely in their performance on verbal adynamia tasks and there was extensive overlap between patient groups (Figure 1). More detailed analysis of patients’ responses on verbal adynamia tasks (Table S5 online) revealed a substantial proportion (ranging from around 25–80%) of “no” [timed-out] responses across subtests in both disease groups. More qualitatively, the PSP/CBS group had a tendency to generate unusual low frequency words (e.g., “plagiarism”, “plantation”). In a separate *post hoc* analysis comparing the patient subgroups with clinical syndromic diagnoses of PSP and CBS, patients with CBS showed more severe impairment of naming skills; however, there were no significant differences between these syndromic subgroups on any verbal generation measure (see Table S7 online).

### 3.3 Effect of dopaminergic modulation

Dopaminergic stimulation in the ON state compared with the OFF state was associated with significantly improved (*p* < 0.05) performance on tests of sentence completion with unconstrained words and sentence generation, with a strong trend to significantly improved phrase generation (*p* = 0.053) and also a trend toward improved sentence completion using constrained words (*p* = 0.08) and improved letter fluency (*p* = 0.09). Dopaminergic state did not affect the frequency of “no response” errors; nor did it affect design fluency or grammaticality indices (see Table S2 online).

### 3.4 Neuroanatomical associations

**Figure 2.**
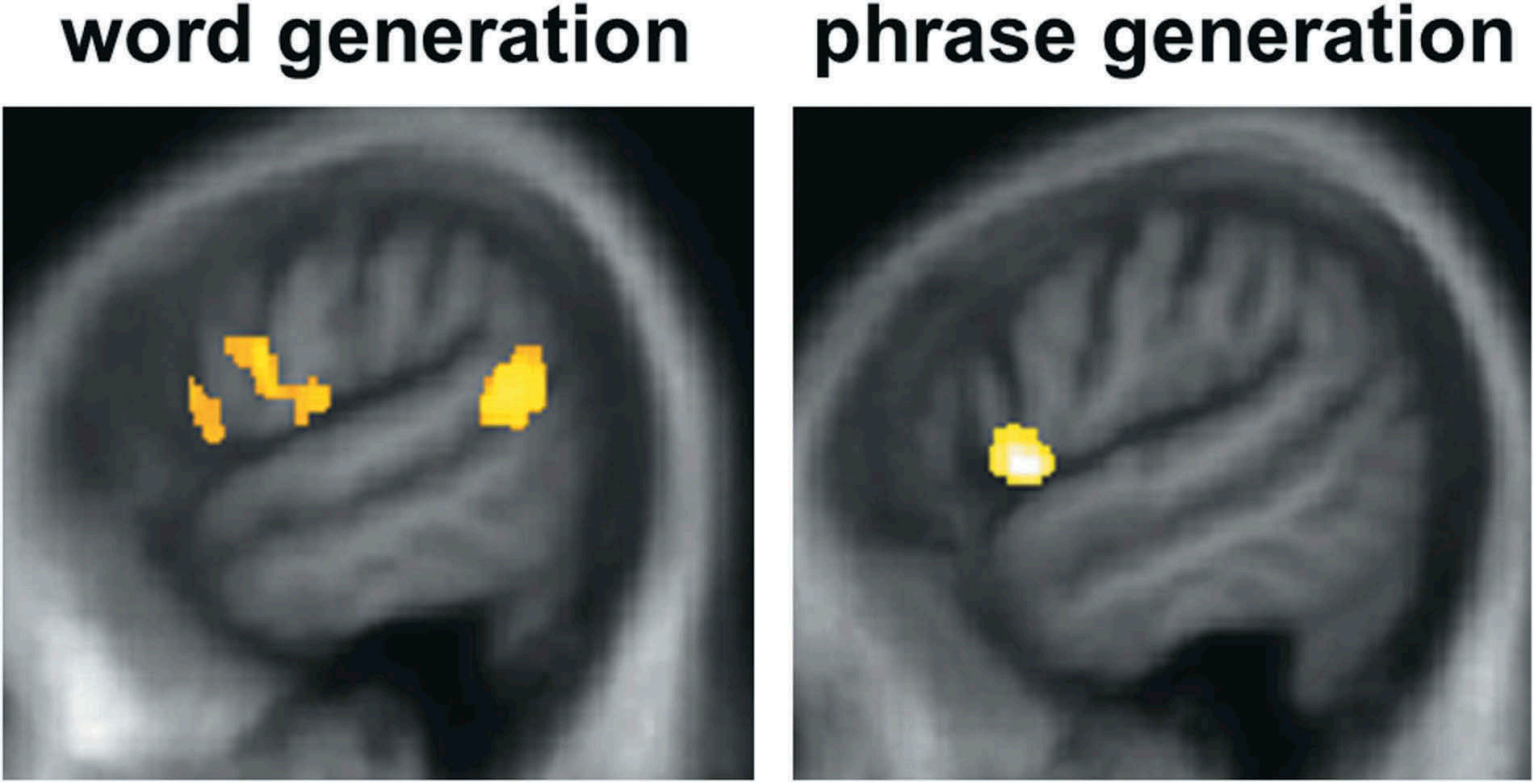
Statistical parametric maps (SPMs) showing regional gray matter atrophy significantly associated with task performance for sentence completion using an unconstrained word (left) or a phrase (right) in the combined patient cohort. SPMs are thresholded at *p* < 0.05 after small volume correction for multiple voxel-wise comparisons in pre-specified small anatomical volumes of interest and displayed on sagittal sections of the left hemisphere from a group mean T1-weighted MR brain template image in Montreal Neurological Institute standard space.

Significant gray matter associations of performance on verbal generation tasks are summarized in Table 3 and statistical parametric maps are presented in Figure 2. No gray matter associations of verbal generation performance were identified at threshold *p* < 0.05_FWE_ over the whole brain. Within the small volumes of interest specified by our prior anatomical hypotheses (Blank et al., 2002; Braun et al., 2001; Wagner et al., 2001), significant neuroanatomical associations of sentence completion task performance were identified: sentence completion with an unconstrained word was positively correlated with gray matter volume in left inferior frontal gyrus and left posterior superior temporal gyrus (*p* < 0.05_FWE_), while sentence stem completion with a phrase was positively correlated with gray matter volume in left inferior frontal gyrus (*p* < 0.05_FWE_). No other significant gray matter associations were identified.

**Table 3.** Neuroanatomical associations of patient performance on verbal adynamia tests.

		Peak (mm)					
Contrast	Region	x	y	z	Cluster (voxels)	*t*-score	*p*-value
Sentence completion: unconstrained word	inferior frontal gyrus	−51	15	0	161	5.73	0.015
	posterior superior temporal gyrus	−62	−46	7	162	5.55	0.016
Sentence completion: phrase	Inferior frontal gyrus	−56	8	1	180	5.26	0.035

All significant regional grey matter associations of performance on verbal generation tasks from the VBM analysis of the combined patient cohort are shown. Coordinates of significant local maxima are in Montreal Neurological Institute standard stereotactic space; all regions are in the dominant (left) hemisphere. Significance has been thresholded at p<0.05 after family-wise error correction for multiple voxel-wise comparisons within pre-specified anatomical regions of interest.

## 4 Discussion

We have shown that patients with parkinsonian syndromes and associated cognitive impairment (PDD and PSP/CBS) have impaired generation of verbal messages (at the level of words and sentences). Deficits of verbal generation were evident after taking associated language deficits and general nonverbal executive performance into account and did not correlate with a measure of nonverbal pattern generation (design fluency). Verbal dynamic performance in the PDD group was modulated by dopaminergic stimulation; this modulation did not extend to nonverbal design fluency or other cognitive functions assessed, suggesting that this may be a relatively specific effect on verbal output. Taken together, our findings are in line with previous evidence for impaired organization of language output in parkinsonism (Petrova et al., 2016) but suggest a more specific limitation of verbal generation. Besides corroborating previous work showing reduced verbal fluency in parkinsonism (Herrera, Cuetos, & Ribacoba, 2012), the profiles of verbal adynamia exhibited by both our patient groups were broadly in keeping with the findings documented in previous detailed case studies of patients with PSP and dynamic aphasia (Esmonde et al., 1996; Robinson et al., 2006; Robinson et al., 2015). However, our study did not differentiate parkinsonian syndromes. While this may in part reflect the inclusion of patients with PD and established cognitive decline (and relatively long clinical duration), verbal adynamia might index a core mechanism of impaired spontaneous thought that is integral to this spectrum of disorders.

Our data in PDD are in line with prior evidence for dopaminergic modulation of other cognitive functions, including letter fluency, verb processing, and various extra-linguistic executive tasks (Costa, Peppe, Dell’Agnello, Caltagirone, & Carlesimo, 2009),(Herrera & Cuetos, 2012; Herrera et al., 2012). Our finding that verbal adynamic deficits go beyond word generation to the generation of connected speech (sentences) suggests that the extent of such deficits in PDD may have been under-recognized, particularly if the severity of any deficit is modified by dopaminergic therapy. Our findings leave open the possibility that particular patient subgroups within the broad disease groupings may show more selective or more prominent verbal generation deficits.

The VBM evidence in the combined patient cohort here suggests a plausible, common neuroanatomical substrate for verbal adynamia in these parkinsonian syndromes, after taking into account the effects of general executive or linguistic compromise (as indexed by WASI Matrices and Graded Naming Test performance). The present evidence implicates core inferior frontal and posterior superior temporal components of the dominant hemisphere language network: similar cortical regions have been linked to propositional speech output in the healthy brain (Wagner et al., 2001) and have been previously shown to be damaged in PDD, CBS, and PSP (Boxer et al., 2006). More specifically, this dominant hemisphere fronto-striatal network (and within this network, left inferior frontal cortex) has been linked to the pathogenesis of dynamic aphasia in detailed case studies of patients with neurodegenerative and focal brain pathologies (Costello & Warrington., 1989; Esmonde et al., 1996; Robinson, 2013; Robinson et al., 2006, 2015; Rohrer et al., 2010). Our study was not equipped to resolve the several candidate mechanisms that have been proposed to underpin dynamic aphasia; however, inferior frontal cortex is likely to be involved in discourse and fluent thought sequencing, as well as verbal response selection, all of which are potentially vulnerable in parkinsonian disorders (Boxer et al., 2006). Our patient cohort showed deficits both on design fluency and verbal generation tasks, as would be anticipated with a domain-general generative impairment (Robinson et al., 2015); however, these verbal and nonverbal deficits were uncorrelated over the cohort while only verbal performance showed dopaminergic modulation, which at least raises the possibility of dissociable processes.

This study has several limitations and we regard the present findings a preliminary rationale for future work. Eliciting truly “spontaneous” speech under laboratory conditions is challenging, however there are neuropsychological instruments (for example, the Western Aphasia Battery) that incorporate speech elicitation prompts; the use of such instruments might amplify the procedure we adopted here and provide a more complete picture of natural verbal generation in parkinsonian disorders. For logistical reasons, ON and OFF states in our PDD group were assessed in fixed order; ideally, this would be randomized (though practice effects are unlikely to have substantially altered our results, given the ON state was assessed first). Further studies in larger cohorts of patients with both PD and other parkinsonian syndromes are required, ideally longitudinally to capture the evolution of any linguistic deficits in relation to overall disease stage and ultimately with histopathological correlation. Larger group sizes would power potentially informative comparisons within disease groupings (for example, CBS versus PSP in the tauopathy spectrum and potentially, different pathological substrates of CBS). It would be particularly informative to assess patients with earlier stage PD prior to onset of clinical cognitive symptoms, in order to establish whether verbal adynamia might provide a novel biomarker for tracking clinical disease evolution. Component cognitive subprocesses that are likely to contribute to verbal adynamia (for example, verbal message creation versus verbal output speed and articulatory programming: Warren et al., 2003) were not distinguished in this study but might be dissected apart in future studies. A complete characterization of the relevant cognitive processes will entail multimodal neuroimaging techniques including functional MRI and tractography to measure connectivity changes in culprit brain networks.

From a clinical perspective, our findings highlight a category of language symptoms that is likely to have been under-recognized. Impaired ability to generate verbal messages might have potentially profound effects on everyday communication and could therefore contribute importantly to overall disease burden in patients with parkinsonism. We hope this work will motivate further assessment of language dysfunction in parkinsonian disorders, in order to determine how verbal adynamia and other linguistic deficits contribute to daily life disability, their potential as disease biomarkers and how such deficits might be treated.

## References

[CIT0001] Arai T., Ikeda K., Akiyama H., Shikamoto Y., Tsuchiya K., Yagishita S., & McGeer P. L. (2001). Distinct isoforms of tau aggregated in neurons and glial cells in brains of patients with Pick’s disease, corticobasal degeneration and progressive supranuclear palsy. *Acta Neuropathologica*, 101, 167–173. 11271372 10.1007/s004010000283

[CIT0002] Armstrong M. J., Litvan I., Lang A. E., Bak T. H., Bhatia K. P., Borroni B., & Weiner W. J. (2013). Criteria for the diagnosis of corticobasal degeneration. *Neurology*, 80, 496–503. 23359374 10.1212/WNL.0b013e31827f0fd1 PMC3590050

[CIT0003] Ashburner J. (2007). A fast diffeomorphic image registration algorithm. *Neuroimage*, 38, 95–113. 17761438 10.1016/j.neuroimage.2007.07.007

[CIT0004] Bak T. H., Hodges J. R., & Thomas H. B. (2008). Coricobasal degeneration: Clinical aspects In: Duyckaerts C. & Litvan I. (eds.), *Handbook of clinical neurology* (Vol. 89, pp. 509–521). Amsterdam: Elsevier. 10.1016/S0072-9752(07)01247-X 18631773

[CIT0005] Baxter D. M., & Warrington E. K. (1994). Measuring dysgraphia: A graded difficulty spelling test. *Behavioural Neurology*, 7, 107–116. 24487323 10.3233/BEN-1994-73-401

[CIT0006] Bhatia K. P., & Marsden C. D. (1994). The behavioural and motor consequences of focal lesions of the basal ganglia in man. *Brain : a Journal of Neurology*, 117, 859–876. 7922471 10.1093/brain/117.4.859

[CIT0007] Blank S. C., Scott S. K., Murphy K., Warburton E., & Wise R. J. (2002). Speech production: Wernicke, Broca and beyond. *Brain : A Journal of Neurology*, 125, 1829–1838. 12135973 10.1093/brain/awf191

[CIT0008] Bloom P. A., & Fischler I. (1980). Sentence completion tasks. *Memory and Cognition*, 8, 631–642. 7219183 10.3758/bf03213783

[CIT0009] Boxer A. L., Geschwind M. D., Belfor N., Gorno-Tempini M. L., Schauer G. F., Miller B. L., & Rosen H. J. (2006). Patterns of brain atrophy that differentiate corticobasal degeneration syndrome from progressive supranuclear palsy. *Archives of Neurology*, 63, 81–86. 16401739 10.1001/archneur.63.1.81

[CIT0010] Braun A. R., Guillemin A., Hosey L., & Varga M. (2001). The neural organization of discourse: An H2 15O-PET study of narrative production in English and American sign language. *Brain : A Journal of Neurology*, 124, 2028–2044. 11571220 10.1093/brain/124.10.2028

[CIT0011] Costa A., Peppe A., Dell’Agnello G., Caltagirone C., & Carlesimo G. A. (2009). Dopamine and cognitive functioning in de novo subjects with Parkinson’s disease: Effects of pramipexole and pergolide on working memory. *Neuropsychologia*, 47, 1374–1381. 19428401 10.1016/j.neuropsychologia.2009.01.039

[CIT0012] Costello A., & Warrington. E. K. (1989). Dynamic Aphasia: The selective impairment of verbal planning. *Cortex; A Journal Devoted to the Study of the Nervous System and Behavior*, 25, 103–114. 2539945 10.1016/s0010-9452(89)80010-3

[CIT0013] Desikan R. S., Segonne F., Fischl B., Quinn B. T., Dickerson B. C., Blacker D., & Killiany R. J. (2006). An automated labeling system for subdividing the human cerebral cortex on MRI scans into gyral based regions of interest. *Neuroimage*, 31, 968–980. 16530430 10.1016/j.neuroimage.2006.01.021

[CIT0014] Emre M., Aarsland D., Brown R., Burn D. J., Duyckaerts C., Mizuno Y., & Dubois B. (2007). Clinical diagnostic criteria for dementia associated with Parkinson’s disease. *Movement Disorders : Official Journal of the Movement Disorder Society*, 22, 1689–1707. quiz 1837. 17542011 10.1002/mds.21507

[CIT0015] Esmonde T., Giles E., Xuereb J., & Hodges J. (1996). Progressive supranuclear palsy presenting with dynamic aphasia. *Journal of Neurology, Neurosurgery, and Psychiatry*, 60, 403–410. 10.1136/jnnp.60.4.403 PMC1073893 8774405

[CIT0016] Gross R. G., McMillan C. T., Chandrasekaran K., Dreyfuss M., Ash S., Avants B., & Grossman M. (2012). Sentence processing in Lewy body spectrum disorder: The role of working memory. *Brain Cogn*, 78, 85–93. 22218297 10.1016/j.bandc.2011.12.004 PMC3265703

[CIT0017] Helm-Estabrooks N., Nicholas M., & Helm S. A. (2000). *Sentence production programme for Aphasia* (2 ed.). Austin, Texas: Pro-Ed.

[CIT0018] Herrera E., & Cuetos F. (2012). Action naming in Parkinson’s disease patients on/off dopamine. *Neuroscience Letters*, 513, 219–222. 22387157 10.1016/j.neulet.2012.02.045

[CIT0019] Herrera E., Cuetos F., & Ribacoba R. (2012). Verbal fluency in Parkinson’s disease patients on/off dopamine medication. *Neuropsychologia*, 50, 3636–3640. 22995942 10.1016/j.neuropsychologia.2012.09.016

[CIT0020] Hoehn M. M., & Yahr M. D. (1967). Parkinsonism: Onset, progression and mortality. *Neurology*, 17, 427–442. 6067254 10.1212/wnl.17.5.427

[CIT0021] Hoglinger G. U., Respondek G., Stamelou M., Kurz C., Josephs K. A., & Lang A. E.; Movement Disorder Society-endorsed (2017). Clinical diagnosis of progressive supranuclear palsy: The movement disorder society criteria. *Movement Disorders : Official Journal of the Movement Disorder Society*, 32, 853–864. 28467028 10.1002/mds.26987 PMC5516529

[CIT0022] Jackson M., & Warrington E. K. (1986). Arithmetic skills in patients with unilateral cerebral lesions. *Cortex; a Journal Devoted to the Study of the Nervous System and Behavior*, 22, 611–620. 3816245 10.1016/s0010-9452(86)80020-x

[CIT0023] Jenkinson M., Beckmann C. F., Behrens T. E. J., Woolrich M. W., & Smith S. M. (2012). Fsl. *Neuroimage*, 62, 782–790. 21979382 10.1016/j.neuroimage.2011.09.015

[CIT0024] Kaplan D. (2001). D-KEFS Executive Function System. San Antonio, Texas: The Psychological Corporation.

[CIT0025] Kaplan E., & Weinthaubs S. G. H. (2001). *Boston naming test* (2nd ed.). Philadelphia, USA: Lippincott Williams & Wilkins.

[CIT0026] Kay J., Lesser R., & Coltheart M. (1992). *Psycholinguistic assessment of language processing in Aphasia*. London: Lawrence Erlbaum.

[CIT0027] Kertesz A., Martinez-Lage P., Davidson W., & Munoz D. G. (2000). The corticobasal degeneration syndrome overlaps progressive aphasia and frontotemporal dementia. *Neurology*, 55, 1368–1375. 11087783 10.1212/wnl.55.9.1368

[CIT0028] Kertesz A., & McMonagle P. (2010). Behavior and cognition in corticobasal degeneration and progressive supranuclear palsy. *Journal of the Neurological Sciences*, 289, 138–143. 19733862 10.1016/j.jns.2009.08.036

[CIT0029] Lange R. T., Chelune G. J., & Tulsky D. S. (2006). Development of WAIS-III general ability index minus WMS-III memory discrepancy scores. *The Clinical Neuropsychologist*, 20, 382–395. 16895854 10.1080/13854040590967586

[CIT0030] Litvan I., Hauw J. J., Bartko J. J., Lantos P. L., Daniel S. E., Horoupian D. S., & Anderson D. W. (1996). Validity and reliability of the preliminary NINDS neuropathologic criteria for progressive supranuclear palsy and related disorders. *Journal of Neuropathology and Experimental Neurology*, 55, 97–105. 8558176 10.1097/00005072-199601000-00010

[CIT0031] McKenna P., & Warrington E. K. (1983). *The graded naming test*. Windsor, Berks: NFER-Nelson.

[CIT0032] Movement Disorder Society Task Force on Rating Scales for Parkinson’s Disease (2003). The Unified Parkinson’s Disease Rating Scale (UPDRS): Status and recommendations. *Movement Disorders*, 18, 738–750. 12815652 10.1002/mds.10473

[CIT0033] Nelson H. E. (1982). *The National Adult Reading Test (NART): Test manual*. Windsor: NFER-Nelson.

[CIT0034] Petrova M., Pavlova R., Zhelev Y., Mehrabian S., Raycheva M., & Traykov L. (2016). Investigation of neuropsychological characteristics of very mild and mild dementia with Lewy bodies. *Journal of Clinical and Experimental Neuropsychology*, 38, 354–360. 26678542 10.1080/13803395.2015.1117058

[CIT0035] Ridgway G. R., Omar R., Ourselin S., Hill D. L., Warren J. D., & Fox N. C. (2009). Issues with threshold masking in voxel-based morphometry of atrophied brains. *Neuroimage*, 44, 99–111. 18848632 10.1016/j.neuroimage.2008.08.045

[CIT0036] Rittman T., Ghosh B. C., McColgan P., Breen D. P., Evans J., Williams-Gray C. H., & Rowe J. B. (2013). The Addenbrooke’s cognitive examination for the differential diagnosis and longitudinal assessment of patients with parkinsonian disorders. *Journal of Neurology, Neurosurgery, and Psychiatry*, 84, 544–551. 10.1136/jnnp-2012-303618 PMC3623037 23303961

[CIT0037] Robinson G., Shallice T., & Cipolotti L. (2006). Dynamic aphasia in progressive supranuclear palsy: A deficit in generating a fluent sequence of novel thought. *Neuropsychologia*, 44, 1344–1360. 16504225 10.1016/j.neuropsychologia.2006.01.002

[CIT0038] Robinson G. A. (2013). Primary progressive dynamic aphasia and Parkinsonism: Generation, selection and sequencing deficits. *Neuropsychologia*, 51, 2534–2547. 24113151 10.1016/j.neuropsychologia.2013.09.038

[CIT0039] Robinson G. A., Spooner D., & Harrison W. J. (2015). Frontal dynamic aphasia in progressive supranuclear palsy: Distinguishing between generation and fluent sequencing of novel thoughts. *Neuropsychologia*, 77, 62–75. 26247320 10.1016/j.neuropsychologia.2015.08.001

[CIT0040] Rohrer J. D., Crutch S. J., Warrington E. K., & Warren J. D. (2010). Progranulin-associated primary progressive aphasia: A distinct phenotype? *Neuropsychologia*, 48, 288–297. 19766663 10.1016/j.neuropsychologia.2009.09.017 PMC2808475

[CIT0041] Rohrer J. D., Paviour D., Bronstein A. M., O’Sullivan S. S., Lees A., & Warren J. D. (2010). Progressive supranuclear palsy syndrome presenting as progressive nonfluent aphasia: A neuropsychological and neuroimaging analysis. *Movement Disorders : Official Journal of the Movement Disorder Society*, 25, 179–188. 20077483 10.1002/mds.22946 PMC4608044

[CIT0042] Spillantini M. G., Schmidt M. L., Lee V. M., Trojanowski J. Q., Jakes R., & Goedert M. (1997). Alpha-synuclein in Lewy bodies. *Nature*, 388, 839–840. 9278044 10.1038/42166

[CIT0043] Wagner A. D., Pare-Blagoev E. J., Clark J., & Poldrack R. A. (2001). Recovering meaning: Left prefrontal cortex guides controlled semantic retrieval. *Neuron*, 31, 329–338. 11502262 10.1016/s0896-6273(01)00359-2

[CIT0044] Warren, J.D., Warren, J.E., Fox, N.C., & Warrington, E.K. (2003). Nothing to say, something to sing: primary progressive dynamic aphasia. *Neurocase*, 9, 140–155. 12925938 10.1076/neur.9.2.140.15068

[CIT0045] Warrington E. K. (1984). *Recognition memory test: Manual*. Berkshire, UK: NFER-Nelson.

[CIT0046] Warrington E. K., & James M. (1991). *The Visual Object and Space Perception Battery (VOSP)*. Bury St. Edmunds, England: Thames Valley Test Co.

[CIT0047] Warrington E. K. M., & Orpwood, L. P. (1998). Single word comprehension: A concrete and abstract word synonym test. *Neuropsychological Rehabilitation*, 8, 2.

[CIT0048] Wechsler D. (1991). *Manual for the Wechsler intelligence scale for children*. San Antonia, Tx: The Psychological Corporation.

[CIT0049] Weiskopf N., Lutti A., Helms G., Novak M., Ashburner J., & Hutton C. (2011). Unified segmentation based correction of R1 brain maps for RF transmit field inhomogeneities (UNICORT). *Neuroimage*, 54, 2116–2124. 20965260 10.1016/j.neuroimage.2010.10.023 PMC3018573

