# Supplementary material for: Verbal adynamia in parkinsonian syndromes: behavioral correlates and neuroanatomical substrate
Source: Neurocase. 2018 Oct 6;24(4):204–12. doi: 10.1080/13554794.2018.1527368 (PMC6234546; doi:10.1080/13554794.2018.1527368)
Supplement: NCS-OA_17-138-File002.docx [file NNCS_A_1527368_SM3308.docx]

**SUPPLEMENTARY MATERIAL**

**Verbal adynamia in parkinsonian syndromes,** by NK Magdalinou et al

**General neuropsychological tests**

**General intelligence.** The National Adult Reading Test (NART) (Nelson, 1982) is commonly used in clinical settings for estimating premorbid intelligence of English-speaking patients with dementia; reading abilities are thought to be spared in the face of cognitive decline associated with dementia. The Wechsler Abbreviated Scale of Intelligence (WASI) (Corporation, 2002) is a rapid measure of the participant’s verbal, non-verbal and general cognitive functioning. It consists of four subtests (i) Vocabulary: measuring word knowledge and verbal concept formation, (ii) Similarities: measuring verbal reasoning and concept formation, (iii) Block design: measuring the ability to analyse and synthesise abstract visual stimuli, nonverbal concept formation, visual perception and organisation of simultaneous processing and learning, (iv) Matrix reasoning: measuring visual information processing and abstract reasoning skills. The first two subtests comprise verbal IQ and the last two performance IQ.

**Episodic memory.** The Recognition Memory Test for faces (Warrington, 1984) presents 50 faces with an orienting question. The participant is then presented with the target paired with an unfamiliar face and asked to judge, which they have seen before. The same procedure is used for the Recognition Memory Test for words.

**Language skills.** The Graded Naming Test (McKenna and Warrington, 1983) requires participants to name line drawings that become increasingly less familiar. The Boston Naming Test (Kaplan et al., 2001) is another widely used assessment; participants are asked to identify pictures in order of increasing difficulty. Concrete and Abstract Word Synonyms tests (Warrington et al., 1998) provide a graded difficulty assessment of verbal comprehension at the single word level. The Psycholinguistic Assessment of Language Processing in Aphasia (PALPA)-55 subtest of receptive grammar (Kay et al., 1992) asks participants to match verbally presented active and passive sentences to one of three pictures. Here we used a shortened 24-item version. The Baxter Spelling Test (Baxter and Warrington, 1994) is a 30-item graded difficulty written spelling test.

**Other cognitive skills.** The Graded Difficulty Arithmetic Test (Jackson and Warrington, 1986) allows participants 10 seconds to perform increasingly difficult mental arithmetic sums, using addition and subtraction. In the Object Decision subtest of the Visual Object and Space Perception battery (Warrington and James, 1991), participants are asked to identify the silhouette of a 75 degree rotated real object from three nonsense silhouettes of similar complexity.

**References**

Baxter D.M., Warrington E.K. (1994). Measuring dysgraphia: a graded difficulty spelling test. *Behavioural Neurology* 7, 107-116.

Psychological Corporation (2002). Wechsler Memory Scale III, Technical Manual. San Antonio; Texas.

Jackson M., Warrington, E.K. (1986). Arithmetic skills in patients with unilateral cerebral lesions. *Cortex*, 22, 611-620.

Kaplan E., Goodglass H, Weintraub S. (2001). Boston Naming Test. Lippincott Williams & Wilkins (2nd edition).

Kay J., Lesser R., Coltheart M. (1992). Psycholinguistic Assessment of Language Processing in Aphasia. London: Lawrence Erlbaum.

McKenna P., Warrington E.K. (1983). The Graded Naming Test. Windsor, Berks: NFER-Nelson.

Nelson H.E. (1982). The National Adult Reading Test (NART): test manual. Windsor, Berks: NFER-Nelson.

Warrington E.K. (1984). Recognition Memory Test: Manual. Windsor, Berks: NFER-Nelson.

Warrington E.K., James M. (1991). The Visual Object and Space Perception Battery (VOSP). Bury St. Edmunds, England. Thames Valley Test Co.

Warrington E.K., McKenna P., Orpwood L. (1998). Single Word Comprehension: A Concrete and Abstract Word Synonym Test. *Neuropsychological Rehabilitation* 8(2), 143 – 154.

**Table S1.** Details of medication use by patients with Parkinson’s disease dementia

| **Patient** | **Medication** (dose/day, orally unless stated otherwise) |
| --- | --- |
| **1** | Sinemet 1250 mg, Entacapone 1000 mg, Rotigotine patch 2 mg, Rasagiline 1 mg, Rivastigmine 9 mg |
| **2** | Stalevo 900mg, Amantadine 300 mg, Pramipexole SR 2.1 mg |
| **3** | Madopar 375 mg, Amantadine 100 mg, Donepezil 10 mg |
| **4** | Madopar 1000 mg, Rasagiline 1 mg, Rivastigmine patch 9.5 mg |
| **5** | Madopar 375 mg, Rivastigmine patch 9.5 mg |
| **6** | Stalevo 1000 mg, Rivastigmine 6 mg |
| **7** | Stalevo 525 mg, Amantadine 200 mg, Donepezil 10 mg |
| **8** | Sinemet 825 mg, Rasgiline 1 mg, Rivastigmine patch 9.5 mg |
| **9** | Stalevo 800 mg, Rasagiline 1 mg, Amantadine 100 mg, Rivastigmine 6 mg |
| **10** | Madopar 750 mg |
| **11** | Madopar 750 mg, Rivastigmine 6 mg |
| **12** | Apomorphine subcutaneous pump 14 ml |
| **13** | Madopar 562.5 mg, Rivastigmine 6 mg |
| **14** | Stalevo 800 mg, Amantadine 100 mg |
| **15** | Sinemet 1000 mg |
| **16** | Stalevo 375 mg, Rivastigmine 3 mg |
| **17** | Madopar 750 mg, Rasagiline 1 mg, Rivastigmine 6 mg |
| **18** | Sinemet 750 mg |

**Table S2.** Effects of dopaminergic medication in patients with Parkinson’s disease:

comparison of performance on verbal adynamia battery in ON versus OFF states

| **Behavioural domain** | **ON-OFF difference** | **p-value** |
| --- | --- | --- |
| **MOTOR STATE** |  |  |
| UPDRS | **12 (6.5,17.5)** | **0.04** |
| **VERBAL GENERATION FUNCTIONS** |  |  |
| Verbal fluency: initial letter | -0.9 (-2.5, 0.5) | 0.09 |
| Sentence completion: constrained word | 0 (-1, 0) | 0.08 |
| Sentence completion: unconstrained word | **-0.5 (-1.5, 0)** | **0.02** |
| Sentence completion: phrase | 0 (0, 1) | 0.053 |
| Sentence generation: from sentence | **-1 (-1.5, 0)** | **0.03** |
| Sentence generation: from picture sequence | **-0.5 (-1.5, 0)** | **0.04** |
| **CONTROL FUNCTIONS** |  |  |
| Nonverbal fluency: design | 0 (-0.5, 1) | 0.27 |
| Grammaticality | -1 (-1.5, 0) | 0.85 |

Data show median (interquartile range) and p-values of comparison between ON and OFF states in the Parkinson’s disease dementia group (n=16). Significant values (p<0.05) are in bold. The transition from ON to OFF states was substantiated by a significant decrease in UPDRS score. UPDRS, Unified Parkinson’s Disease Rating Scale.

**Table S3.** Stimuli used in verbal generation tasks

**Battery A**

**SENTENCE COMPLETION USING ONE CONSTRAINED WORD**

1. He loosened the tie around his…
2. She dried herself with a…
3. He put the shoe on his left…
4. The horse jumped over the…
5. He took a photograph with his…
6. She watched the programme on the…
7. The girl was milking the…
8. They went to catch a double-decker…
9. He locked the door using his…
10. He read the headlines in the…

**SENTENCE COMPLETION USING ONE UNCONSTRAINED WORD**

1. Jack went to the shop to buy a…
2. The boy walked past the…
3. Mr Smith went into the…
4. The woman read the…
5. He opened the…
6. The whole school went on a trip to the…
7. Mr Jones forgot his…
8. The old man stood in line at the…
9. For our holiday we went to…
10. The colour of the door was…

**COMPLETION OF SENTENCE STEM USING A PHRASE**

1. The children were…
2. The sun disappeared…
3. The music was so…
4. He went to the factory where…
5. John just managed…
6. The woman on the beach…
7. Sally and Peter were…
8. I was not able…

**GENERATION OF SENTENCE FROM A SENTENCE CONTEXT**

1. Joe had fallen and twisted his ankle.
2. The children went down to the beach.
3. Having missed the bus we decided to walk home.
4. The cricket match was very exciting.
5. The storm had caused havoc.
6. The car would not start.
7. We caught the plane from Heathrow in the morning.
8. The policeman signalled the traffic to stop.

**Battery B**

**SENTENCE COMPLETION USING ONE CONSTRAINED WORD**

1. At night, the old woman locked the…
2. The gambler had a streak of bad…
3. They left the dirty dishes in the
4. The bill was due at the end of the…
5. They sat together without speaking a single…
6. Fred realised the old house was up for…
7. Her shoes were too small, they were the wrong…
8. Jim had rehearsed his speech several times, he knew it by…
9. To keep the dogs out of the yard, he put up a…
10. She went to the salon to colour her…

**SENTENTCE COMPLETION USING ONE UNCONSTRAINED WORD**

1. John poured himself a…
2. The hunter shot and killed a large…
3. He was soothed by the gentle…
4. Rushing out, he forgot his…
5. Harry chose not to join the…
6. He went upstairs to fetch a…
7. Yesterday they canoed down the…
8. On his way to work he saw a…
9. Don’t believe everything you…
10. The students felt the test was too…

**COMPLETION OF SENTENCE STEM USING A PHRASE**

1. You could count on Jonathan...
2. His view was blocked…
3. While skiing, Jim…
4. I don’t know why…
5. A direct attack failed, so…
6. She dropped a glass and…
7. Tom could never tell a…
8. The surgeon tried vainly…

**GENERATION OF SENTENCE FROM A SENTENCE CONTEXT**

1. Not even the cast liked the play.
2. Paul has always wanted to be an artist.
3. He crept into the room without a sound.
4. She called her husband at his office.
5. All the guests had a very good time.
6. The lawyer feared that his client was lying.
7. Jack bet all he had on the last race.
8. He wondered if the storm had caused much damage.

**GENERATION OF SENTENCE FROM A PICTURE SEQUENCE (see Figure S1)**

Examples of incorrect responses on verbal generation tasks: Constrained word completion, *She went to the salon to colour her… husband;* Unconstrained word completion, *John poured himself a… man;* Phrase generation from a phrase, *Tom could never tell a… time****;*** Sentence generation from a sentence, *[Paul has always wanted to be an artist] But has never had the chance to learn.*

**Table S4**. Healthy control group performance on neuropsychological stimulus sets A and B

| **Neuropsychological test** | **Set A** | **Set B** | **Difference A vs B** | **p-value** |
| --- | --- | --- | --- | --- |
| Verbal fluency: initial letter (in 60 secs) | 15 | 15.4 | 0 (-1, 1.5) | 0.72 |
| Sentence completion:  constrained word (/10) | 10 | 10 | 0 (0,0) | * |
| Sentence completion:  unconstrained word (/10) | 9.9 | 10 | 0 (0,0) | 0.32 |
| Phrase completion (/8) | 8 | 7.9 | 0 (0,0) | 0.32 |
| Sentence generation:  from sentence (/8) | 7.5 | 7.3 | 0 (-0.5, 0) | 0.59 |
| Sentence generation:  from picture sequence (/6) | 6 | 5.8 | 0 (0-0) | 0.16 |
| Nonverbal fluency: design (in 60 secs) | 7.9 | 7.6 | -0.5 (-1.5, 0) | 0.11 |
| Expressive grammar (/12) | 12 | 12 | 0 (0-0) | * |

Raw scores on both set A and set B batteries are shown (maximum subtest scores are indicated (in parentheses); data values are given as median (interquartile ranges). P-values are given for task comparisons. *unable to assess comparisons as scores were

identical in sets A and B.

**Table S5.** Analysis of errors made by participant groups on verbal generation tasks

| **Test** | **Output type** | **HC**  **n=19** | **PDD**  **n=18** | **PSP/CBS**  **n=11** |
| --- | --- | --- | --- | --- |
|  |  | Mean (range) | Mean (range) | Mean (range) |
| Sentence completion:  constrained word  (/10) | All errors | 0 | 0.3 (0 - 2) | 0.6 (0 - 2) |
|  | No response | 0 | 0.2 (0 - 2) | 0.2 (0 - 2) |
|  | Wrong response | 0 | 0.2 (0 - 2)^a^ | 0.4 (0 - 1)^aa^ |
| Sentence completion:  unconstrained word  (/10) | All errors | 0.3 (0 - 2) | 0.9 (0 - 2) | 0.9 (0 - 4) |
|  | No response | 0 | 0.6 (0 - 2) | 0.6 (0 - 2) |
|  | Wrong response | 0.3 (0 - 2) | 0.3 (0 - 2)^b^ | 0.3 (0 - 2)^bb^ |
| Sentence completion:  phrase  (/8) | All errors | 0 | 1.0 (0 - 2) | 1.2 (0 - 5) |
|  | No response | 0 | 0.7 (0 - 2) | 0.9 (0 - 4) |
|  | Wrong response | 0 | 0.3 (0 - 1)^c^ | 0.3 (0 - 1)^cc^ |
| Sentence generation:  from sentence  (/8) | All errors | 0.4 (0 - 3) | 2.8 (0 - 5) | 3.3 (0 - 6) |
|  | No response | 0 | 0.8 (0 - 3) | 0.9 (0 - 3) |
|  | Wrong response | 0.4 (0 - 3) | 2.0 (0 - 5)^d^ | 2.4 (0 - 6)^dd^ |
| Sentence generation:  from picture sequence  (/6) | All errors | 0.05 (0 - 1) | 2.2 (0 - 4) | 1.6 (0 - 5) |
|  | No response | 0 | 0.8 (0 - 4) | 1.3 (0 - 4) |
|  | Wrong response | 0.05 (0 - 1) | 1.4 (0 - 4)^e^ | 0.3 (0 - 3)^ee^ |

The Table quantifies types of errors produced as a mean frequency of errors of that type (either wrong responses or no responses) across that participant group, with the range in number of errors made by individual participants in parentheses; these data correspond to the plots shown in Figure 1. ‘Wrong responses’ in the patient groups, comprised the following error types (proportion of total errors on the relevant test, made by that patient group): a, 50% agrammatic - 50% semantic; aa, 100% agrammatic; b, 100% agrammatic; bb, 50% agrammatic - 50% semantic; c, 100% agrammatic; cc, 100% agrammatic; d, 40% agrammatic – 60% semantic; dd, 80% agrammatic - 20% semantic; e, 50% picture description - 50% semantic; ee, 30% picture description – 70% semantic. See Figure S1 legend for examples of error types. HC, healthy control group; PDD, patient group with Parkinson’s disease dementia; PSP/CBS, patient group with progressive supranuclear palsy / corticobasal syndrome

**Table S6**. Verbal generation test performance compared between study groups

| **Cognitive task** | **Comparison** | **Difference means** | **95% CI** |
| --- | --- | --- | --- |
| Letter fluency | PDD vs HC | **-5.0** | **-8.8, -1.2** |
|  | PSP/CBS vs HC | **-8.3** | **-12.7, -4.5** |
|  | PDD vs PSP/CBS | -3.3 | -6.9, 0.5 |
| Category fluency | PDD vs HC | **-5.2** | **-8.3, -1.7** |
|  | PSP/CBS vs HC | **-7.7** | **-11.7, -4.8** |
|  | PDD vs PSP/CBS | -2.6 | -6.6, 0.35 |
| Sentence completion:  constrained word | PDD vs HC | 0.2 | 0.0, 0.6 |
|  | PSP/CBS vs HC | -0.4 | -1.1, 0.0 |
|  | PDD vs PSP/CBS | **-0.6** | **-0.2, -0.1** |
| Sentence completion:  unconstrained word | PDD vs HC | 0.0 | -0.8, 0.8 |
|  | PSP/CBS vs HC | -0.4 | -1.5, 0.7 |
|  | PDD vs PSP/CBS | -0.4 | -1.5, 0.7 |
| Sentence completion:  phrase | PDD vs HC | 0.2 | -0.1, 0.1 |
|  | PSP/CBS vs HC | -0.3 | -1.3, 0.1 |
|  | PDD vs PSP/CBS | -0.5 | -1.5, 0.0 |
| Sentence generation:  from sentence | PDD vs HC | **-2.4** | **-4.1, -0.8** |
|  | PSP/CBS vs HC | **-2.4** | **-4.1, -0.8** |
|  | PDD vs PSP/CBS | 0.4 | -1.3, 1.9 |
| Sentence generation:  from picture sequence | PDD vs HC | **-0.9** | **-3.0, -0.5** |
|  | PSP/CBS vs HC | **-0.4** | **-2.8, -0.4** |
|  | PDD vs PSP/CBS | 0.5 | -1.0, 1.8 |

Data for group mean differences with 95% confidence intervals (CI) are presented adjusted for nuisance covariates of gender, performance on WASI Matrices (general executive function) and adynamia linguistic control task performance (noun retrieval, expressive grammar). Significant differences in mean scores (p<0.05) are indicated in bold. Due to use of bootstrapping, p values are not provided; statistical significant at p<0.05 can be inferred from 95% CI. GNT, Graded Naming Test; HC, healthy control group; PDD, patients with Parkinson’s disease dementia; PSP/CBS, patients with progressive supranuclear palsy / corticobasal syndrome

| **Characteristic** | **PSP** | **CBS** |
| --- | --- | --- |
| **GENERAL** |  |  |
| Number (female: male) | 7 (5:2) | 4 (2:2)* |
| Age (years) | 71 (6.5) | 66 (8.2) |
| Handness (right: left) | 7:0 | 4:0 |
| Education (years) | 15 (3.6) | 14.8 (2.9) |
| **CLINICAL** |  |  |
| Disease duration (y) (median (IQR)) | 2 (2-7) | 3.5 (3-4.5) |
| MMSE | 28 (26-29) | 26 (22-28) |
| HY | 3 (1) | 2.9 (1) |
| UPDRS | 29.4 (11) | 31.5 (6) |
| **VERBAL GENERATION FUNCTIONS** |  |  |
| Sentence completion: constrained word (/10) | 10 (9-10) | 9 (8.5-9.5) |
| Sentence completion: unconstrained word (/10) | 10 (9-10) | 8.5 (8-9.5) |
| Sentence completion: phrase (/8) | 8 | 7.5 (7-8) |
| Sentence generation: from sentence (/8) | 5 (4-8) | 4 (3.5-5.5) |
| Sentence generation: from picture sequence (/6) | 6 (5-6) | 5 (4-5) |
| **CONTROL FUNCTIONS** |  |  |
| Fluency: Initial letter (in 60 secs) | 9 (2-10) | 5.5 (3-7.5) |
| Fluency: Design (in 60 secs) | 4.5 (4-5) | 4 (3-5) |
| Naming: GNT (/30) (mean, SD) | 22 (20-24) | 7 (4-10)* |
| Naming: BNT (/30) (mean, SD) | 25 (1.8) | 18.3 (9.6) |
| Naming**:** Verbs (/20) | 19.5 (19-20) | 15 (14-19)* |
| Expressive grammar (/12) | 11 (11-12) | 11.5 (11-12) |
| Receptive Grammar-PALPA55 (/24) | 23 (23-24) | 15 (15-21) |

**Table S7.** Summary comparison of PSP and CBS subgroups

For neuropsychological tests, maximum scores are indicated (in parentheses) and values are given as median (interquartile range, IQR) unless otherwise indicated. *significant difference between disease groups. BNT, Boston Naming Test; CBS, patients with corticobasal syndrome; GNT, Graded Naming Test; HY, Hoehn and Yahr score; MMSE, Mini-Mental State Examination score; PALPA, Psycholinguistic Assessment of Language Processing in Aphasia; PSP, patients with progressive supranuclear palsy; SD, standard deviation; UPDRS, Unified Parkinson’s Disease Rating Scale.


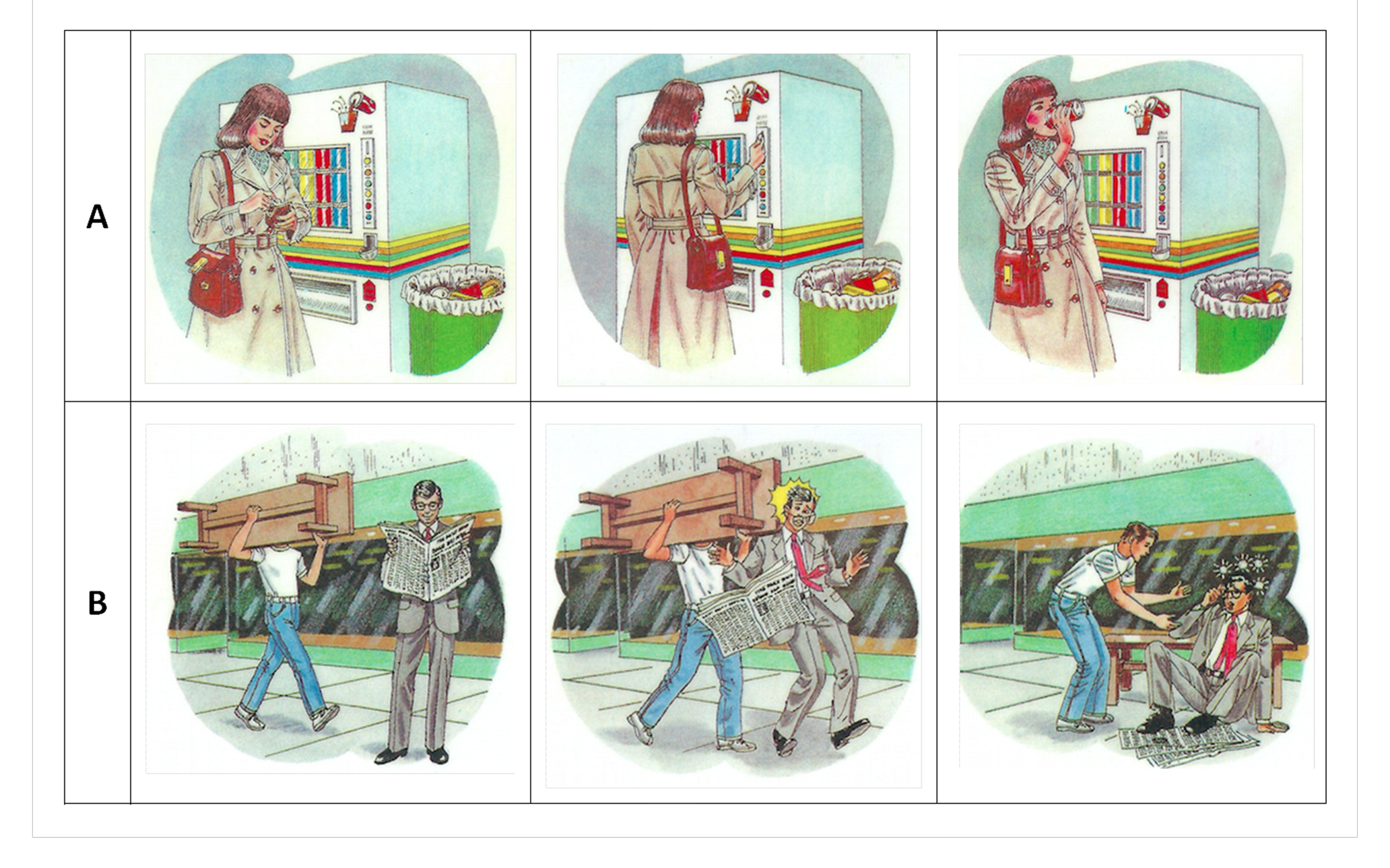


**Figure S1.** Examples of stimuli used to assess generation of a sentence from a picture sequence (adapted from Wechsler, 1991). Representative trials from batteries A and B are shown. Examples of correct responses: **A**, *She is going to put the can in the waste bin*; **B**, *He is going to ask the man he’s knocked over how he is feeling*. Example of incorrect responses: **A**, *Jane finds some change in the meter* [semantic]; **B***, He is got to help him up* [agrammatic]. Simply describing the picture content (rather than continuing the story) or failing to make any response by 20 seconds were also counted as errors.
